# Impact of cooking on the protein quality of Russet potatoes

**DOI:** 10.1002/fsn3.3734

**Published:** 2023-10-03

**Authors:** Taryn Bailey, Adam J. Franczyk, Erin M. Goldberg, James D. House

**Affiliations:** ^1^ Department of Food and Human Nutritional Sciences University of Manitoba Winnipeg Manitoba Canada; ^2^ Richardson Centre for Food Technology and Research University of Manitoba Winnipeg Manitoba Canada

**Keywords:** cooking method, in vitro protein digestibility, protein digestibility‐corrected amino acid score, protein quality, russet potato, true fecal digestibility

## Abstract

Despite being low in crude protein, on a fresh weight basis, given their overall contribution to the North American diet, potatoes contribute approximately 2%–4% of the population's protein intake. However, the quality of the protein remains ill‐defined. To that end, Russet potatoes were secured and subjected to various cooking conditions (raw [control], boiled, baked, microwaved, and fried [3, 6, and 9 min]) to determine the impact of cooking method on protein quality, as determined by amino acid score (AAS) and indices of in vivo true fecal protein digestibility (TFPD%; rodent bioassay) and in vitro protein digestibility (pH‐drop, pH‐Stat, and simulated gastrointestinal digestion both static and dynamic). The AAS of raw Russet potatoes was 0.67 ± 0.01, with histidine being the limiting AA. Frying led to a significant reduction in the AAS, however, other cooking methods yielded similar results to the raw control. The TFPD% of raw potato was low (40.5% ± 3.9%) and was significantly enhanced to over 80% with all cooking methods. Similar patterns were observed with all in vitro measures, however, all methods yielded higher values for the raw control samples. Final protein digestibility‐corrected AAS (PDCAAS; product of AAS and TFPD%) values ranged from 0.27 (raw) to a high of 0.57 (boiled), with cooked values being comparable to other plant‐based protein sources, including grains, and some nuts and pulses. In vitro PDCAAS values followed similar trends. This study defined the protein quality of cooked Russet potatoes and provides data for use in defining the quality of total protein consumed in the North American diet.

## Graphical abstract

The protein digestibility‐corrected amino acid score (PDCAAS) of Russet potatoes subjected to various cooking methods ranged between 0.27 (raw) and a high of 0.57 (boiled), with cooked values being comparable to other plant‐based protein sources, including grains, and some nuts and pulses. In vitro PDCAAS values followed similar trends. This study defined the protein quality of cooked Russet potatoes and provides data for use in defining the quality of total protein consumed in the North American diet.

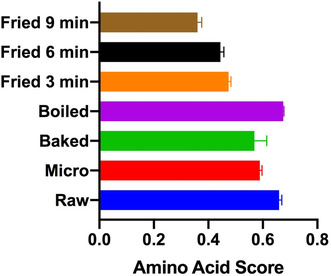

## 1 INTRODUCTION

Potatoes are grown globally and represent the third most important food crop, behind wheat and rice (Birch et al., 2012). Belonging to the *Solanaceae* family, with the Latin name *Solanum tubersum*, there are over 5000 varieties of potatoes that are cultivated worldwide (Lutaladio et al., 2009). The global acceptance of potatoes as a food source stems from their versatility in various cuisines and their relative nutritional profile. Recognized as a vegetable (starchy vegetable) in both the United States (U.S. Department of Agriculture & U.S. Department of Health and Human Services, 2022) and Canadian (Health Canada, 2019) dietary guidelines, potatoes fit within healthy eating patterns, including those where the focus is placed on increasing the intake of plant‐based foods. As recent interest has focused on plant‐based sources of protein, the contribution of potatoes to protein intake is not well‐defined, likely due to the fact that their overall crude protein content is only 2%–4% by weight in raw potato, with these values subject to change due to cooking method, varietal selection, and potentially agronomic practices. Given the intake of potatoes in the Canadian population (Polsky & Garriguet, 2020) and the total protein intake (Fabek et al., 2021), it is estimated that potatoes can provide approximately 2%–4% of total daily protein intake. What remains ill‐defined is the quality of the protein contained within this starchy vegetable.

In order to make claims in both the United States and Canada related to the protein content of a food, the quality of the protein must be defined. As positioned by the FAO in 1991 (FAO/WHO, 1991) and again in 2013 (FAO/WHO, 2013), protein quality is generally defined by two key components: (1) the amino acid composition of the food in relation to the reference pattern of amino acid requirements for humans; and (2) the extent to which the food protein is digested and the constituent amino acids made available for absorption and utilization for productive purposes. The official method used by the U.S. FDA to determine protein quality includes the use of the protein digestibility‐corrected amino acid score (PDCAAS) method (US Food and Drug Administration, 2022). The use of PDCAAS is now accepted by Health Canada for the determination of the protein rating needed to substantiate protein content claims (Health Canada, 2020). Given the requirement for data related to protein quality of potatoes, there is a need to determine the impact of various cooking methods on both the amino acid composition and digestibility of potato protein.

Information about the protein quality of whole potatoes is lacking in the literature, along with data as to the degree to which at‐home cooking methods impact protein quality. As a major potato varietal, Russet potatoes are often subjected to multiple processing methods, particularly home‐cooking methods such as boiling, baking, microwaving, and deep frying. Thermal processing has been shown to impact the quality of the protein found in various foods, including pulses (Nosworthy et al., 2018a, 2020; Stone et al., 2021) and cereals (Han et al., 2019; Wang et al., 2020), however, there is limited information as to the impact of processing on potato protein quality. Recent data have focused on potato protein isolates (Donadelli et al., 2019), which have been shown to have high AASs and digestibility. However, data on whole potatoes are lacking. As such, the overall objective of the current research was to determine the impact of commonly used cooking methods on the protein quality of Russet potatoes. In this study, PDCAAS was measured using digestibility values determined via four in vitro methods: pH‐drop, pH‐stat, and simulated gastrointestinal digestion methods, both static and dynamic. Also, the official method for determining true fecal protein digestibility (TFPD), using a rodent bioassay, was employed in order to generate PDCAAS values as stipulated by the US Food and Drug Administration (2022).

## 2 EXPERIMENTAL PROCEDURES

### 2.1 Statement on animal ethics

All animal procedures received approval from the University of Manitoba's Institutional Animal Care Committee, which utilized the appropriate guidelines established by the Canadian Council on Animal Care (2018).

### 2.2 Chemicals

All chemicals and reagents were purchased from Sigma (Oakville, ON, Canada). Diet ingredients were purchased from Dyets, Inc. (Bethlehem, PA, USA). High nitrogen casein (>85% protein) used as a standard in the in vitro protein digestibility assays was also purchased from Dyets, Inc.

### 2.3 Sample procurement and processing

Idaho Russet potatoes were secured from a local farmer (location NW15‐7‐5) from an October 2018 harvest, as facilitated by Kroeker Farms (Winkler, Manitoba, Canada). Potatoes were stored at 4°C until processed.

Four cooking methods were used in this study, each with three independent replicates: BAKED, boiled, microwaved, or fried. Raw potato was used as the control. As no standardized methods could be found for the respective cooking methods, trial runs were performed to establish methods that allowed for potatoes to be fully cooked, in a manner that mimics common home cooking methods. Once all cooking methods were performed, potatoes were mixed in an industrial Hobart mixer at speed 2 to yield a homogeneous mixture. All weights pre‐ and post‐cooking were recorded to determine the fresh weight composition of processed potatoes. Specifics for the individual cooking treatments are found below.

#### 2.3.1 Raw potato (control)

Whole potatoes (4 kg per replicate) were peeled and submerged in cold water followed by processing using a commercial food mill (Bird, mini‐22 mini mixer grinder, 20 kg capacity, *L* = 89 cm, *W* = 64 cm, *H* = 132 cm). Further processing was performed as described below.

#### 2.3.2 Microwaved

Unpeeled potatoes were pierced with a fork, then cooked in a commercial microwave (20 cm^3^; 700 watts), individually, for 5 min on the highest setting. Once the entire 4 kg replicate batch was cooked, potatoes were removed and allowed to cool to room temperature. Once cool, the entire pith was removed, pooled within replicate, and then processed as described below.

#### 2.3.3 Baked

Unpeeled potatoes were pierced with a fork, then the entire batch (4 kg per replicate) was placed in the middle of a convection oven (Moffat ECP‐3) at 204°C for 55 min to ensure thorough cooking. Once cooked, potatoes were cut lengthwise and allowed to cool. After cooling, the pith of the potato was removed, pooled within replicate, and then processed as described below.

#### 2.3.4 Boiled

Whole potatoes were peeled, cut into 2 × 2 × 2 cm cubes, then placed into a steam kettle (Groen, D‐10, 37.9 L) with boiling water, sufficient to completely submerge the potatoes plus an additional 7.5 cm. Following cooking for 12 min, potatoes were removed, cooled to room temperature, and then processed as described below.

#### 2.3.5 Fried

Potatoes were peeled and cut into 1 × 1 × 5 cm pieces using a commercial manual French fry cutter. After cutting, potato strips were blotted dry and cooked in batches at a ratio of 1:7 of potato: oil (100% high‐oleic canola oil), at 190°C. Potatoes were cooked for 3, 6, or 9 min (Garland 80–03, oil capacity 40 L). Following cooking, the fried potato samples were cooled and further processed as described below.

Following the respective cooking treatment, each replicate of the cooked and cooled potatoes (4 kg) was individually combined in a Hobart mixer (5 min) to ensure homogeneity. All mixed samples were stored in 15.24‐cm‐diameter disposable aluminum pie dishes covered with perforated plastic lids, with each dish containing between 200 and 500 g of product, depending on the cooking method. Samples were frozen at −18°C and then frozen samples were subjected to freeze drying using a SP VirTis Genesis 25 L freeze drier (−70°C) until completely dry.

### 2.4 Analytical procedures

Dried potato samples were milled using a Retsch Ultra Centrifugal Mill ZM 200, with a 0.75 mm sieve, and the total content of each replicate was stored in airtight freezer bags at −18°C until analyzed.

Prior to analysis, fried potato samples were subjected to defatting using hexane (Soxhlet system: Electrothermal CMV12C) in order to reduce the fat content to <10%. Crude protein (CP: *N* × 6.25; AOAC 990.03(M); AOAC International, 2023), crude fat (AOCS AM5‐04; American Oil Chemists' Society, 2020), and dry matter (AOAC 930.15; AOAC International, 2023) of all dried samples were determined by an external laboratory (Central Testing Laboratory), an ISO accredited lab by Standard Council of Canada, using approved analytical procedures. Nitrogen content of both test articles, final diets and rat feces, were determined using the Dumas combustion technique (AOAC 990.03(M)). The use of the 6.25 nitrogen conversion factor, although likely not accurate for most plant‐based foods (Hou et al., 2019), was based on the requirements established by regulatory bodies for the determination of PDCAAS.

The total amino acid profiles of the potato samples were determined according to methods published previously (House et al., 2019). In brief, the AOAC Official Method 982.30 acid hydrolysis was used to determine the contents of most amino acids, based on the use of 6N hydrochloric acid hydrolysis procedure over a 24‐h period. This method leads to the deamidation of both asparagine and glutamine, so these two amino acids are included within the total aspartate and glutamate values reported, as per standard convention within national nutrient databases. Methionine and cysteine were determined by pre‐oxidation with performic acid followed by acid hydrolysis following the AOAC official method 45.4.05. Tryptophan was determined using alkaline hydrolysis, using the protocol established by the International Organization for Standardization (2016). All amino acids were derivatized and separated using the AccQ‐Tag Ultra Chemistry System (Waters, Ltd.) on a Shimadzu UPLC System (AccQ‐Tag Ultra C18, 1.7 μm column), with SIL‐20AC autosampler. Amino acid contents were expressed using their respective hydrated molecular weights. Data were expressed on an “as consumed” and “dry matter” basis.

### 2.5 Amino acid scores

The AAS for each of the five samples, and the reference protein casein, were determined according to the FAO protocol (FAO/WHO, 1991), using the reference amino acid requirement pattern of 2‐ to 5‐year‐old school children. The AAS was calculated by dividing each essential amino acid (mg/g protein) by its respective reference pattern amount, where the reference values (mg/g protein) were as follows: histidine, 19; isoleucine, 28; leucine, 66; lysine, 58; methionine & cysteine, 25; phenylalanine and tyrosine, 63; threonine, 34; tryptophan, 11; valine, 35. The final AAS was determined by selecting the lowest determined ratio.

### 2.6 In vivo true fecal protein digestibility

In vivo TFPD was determined using a rodent bioassay, following the AOAC Official Method 991.29 (AOAC International, 2023). In short, semi‐purified diets were formulated to contain 10% crude protein (*N* × 6.25), where all of the protein was provided by the respective potato treatment. Vitamins and minerals were added via a premix (AIN‐93 formulations; Harlan Teklad) to meet the nutritional requirements of the laboratory rat. Weanling male laboratory rats (*n* = 10 per treatment) were housed individually in suspended wire‐bottom cages. Rats were acclimatized over a 4‐day period, in which they were fed 15 g/day with water available for ad libitum consumption. Once acclimatized, a 5‐day balance period was initiated, which included the measurement of daily food intake and total fecal collection. At the end of the study, the total fecal output was collected, freeze‐dried, and analyzed for dry matter and nitrogen content. TFPD% was calculated using the following formula:

$$\begin{array}{c} \text{TFPD}\%=\left(\text{nitrogen intake}-\left(\text{fecal nitrogen loss}-\text{metabolic nitrogen loss}\right)\right)/\\ \left(\text{nitrogen intake}\right)\times 100. \end{array}$$

Metabolic nitrogen loss was determined as the fecal nitrogen produced per gram of diet consumed by rats consuming a protein‐free diet. For the in vivo assessment of TFPD, the 6‐min fried sample of potato was chosen as the representative frying treatment.

### 2.7 In vitro protein digestibility assessment

Four independent methods were employed to determine the in vitro protein digestibility of the potato samples arising from the different treatment protocols, including three static methods (pH‐Drop; pH‐Stat; and simulated gastrointestinal digestion) and one dynamic model (TIM‐1).

The pH‐Drop method followed the protocol developed by Hsu et al. (1977), as modified by Tinus et al. (2012). The pH‐Drop method monitors the change in pH of a protein solution as a function of amino acid release during digestion. The protein from each treatment, in triplicate, was weighed to 62.5 ± 1 mg and added to 10 mL of Milli‐Q water with subsequent heating to 37°C. To this solution was added a multienzyme cocktail consisting of 3.1 mg/mL chymotrypsin (bovine ≥40 units/mg protein), 1.6 mg/mL trypsin (porcine pancreas 13,000–20,000 BAEE units/mg protein), and 1.3 mg/mL protease (*Streptomyces griseus* ≥15 units/mg solid), prepared in 10 mL Milli‐Q water and heated to 37°C. Both the test ingredient and the cocktail were brought to a pH of 8.0 ± 0.05 with either 1 M NaOH or 1 M HCl and allowed to solubilize for 1 h. The pH‐Drop was initiated when 1 mL of the enzyme cocktail was added to the potato treatment solution. The initial pH was recorded before the pH drop began, with subsequent recordings every 30 s for a total of 10 min. The in vitro pH‐Drop protein digestibility (IV‐PHDPD) was calculated as:

$$\mathrm{IV}-\text{PHDPD}\%=65.66+\left(18.10\times {\mathrm{\Delta pH}}_{10\min}\right)$$

where ΔpH_10min_ represents the change in pH over the 10‐min incubation period from the initial pH of 8.0.

The pH‐Stat procedure followed the method published by Pedersen and Eggum (1983). In brief, 62.5 mg of protein equivalents (*N* × 6.25) derived from test articles were incubated in an enzymatic cocktail containing 1.61 mg/mL trypsin (porcine pancreas 13,000–20,000 BAEE units/mg protein), 3.96 mg/mL chymotrypsin (bovine pancreas ≥40 units/mg protein), and 2.36 mg/mL protease (*S. griseus* ≥15 units/mg solid), prepared in 10 mL of Milli‐Q water and heated at 37°C. Both the sample protein and the enzyme cocktail were brought to a pH of 8 ± 0.5 with 1 M NaOH or HCl, following a 60 min pH stabilization process. Following the addition of enzymes, pH was held at 7.98 using 0.1 N NaOH, and the volume of 0.1 N NaOH was used to hold the pH recorded. The in vitro pH‐Stat protein digestibility (IV‐PHSPD) was calculated using the formula:

$$\mathrm{IV}-\text{PHSPD}\%=76.14+\left(47.77\times \left(\sum 0.\text{1N NaOH}\right)\right).$$

The simulated static gastrointestinal digestion protocol for measuring in vitro protein digestibility followed the method of Boisen and Ferna ´ndez (1995), with modified pH (Boisen, 2007), protein precipitation and centrifugation steps (Montoya et al., 2008), the subsequent filtration, reduction in sample volume, as well as the addition of an amino acid hydrolysis step and determination of α‐amino nitrogen content via an o‐phthaldialdehyde (OPA) method (Church et al., 1983). Full details of the method are available in Franczyk (2018). The in vitro static gastrointestinal protein digestibility (IV‐SGPD) was calculated as:

$$\begin{array}{c} \mathrm{IV}-\text{SGPD}\%=\left(\text{Initial Digest Nitrogen}\right)/\\ \left(\text{Final Digest Nitrogen}\right)\times 100. \end{array}$$

A final method for determining overall protein digestibility utilized the dynamic computer‐controlled gastrointestinal digestion model (TIM‐1; Minekus, 1998). This model simulates human digestion within the gastric and small intestinal (duodenum, jejunum, and ileum) regions. For this model, 3 g of protein (from each cooking replicate) was added to the TIM‐1 stomach. The 6‐h digestion protocol followed that previously published (Minekus, 1998). After the run time was complete, the total efflux was collected, and freeze‐dried for nitrogen output analysis. The remaining material in the TIM‐1 was collected, frozen at −18°C, freeze‐dried, and retained as residue for analysis of total nitrogen via the Dumas method. In vitro TIM‐1 protein digestibility (IV‐TIMPD) was determined as:

$$\begin{array}{c} \mathrm{IV}-\text{TIMPD}\%=\left(\text{input protein}-\text{output final protein}\right)/\\ \left(\text{input protein}\right)\times 100. \end{array}$$

The input protein was calculated as the initial mass of protein with subtractions for the protein contained in the residue and the protein found from other nonfood sources within the residue (blank). The output final protein was derived from the final protein content in the efflux with subtractions for the nonfood source proteins (blank) in the efflux.

For all in vitro digestibility assays, in addition to the potato treatments, a casein standard was included, in triplicate, for standardization of all assays to a casein digestibility coefficient of 100%.

### 2.8 Protein digestibility‐corrected amino acid scores

For final in vivo and in vitro, PDCAAS values were calculated as the product of the AAS for each potato treatment and the corresponding protein digestibility coefficient as follows:

$$\begin{array}{c} \text{PDCAAS}\%=\text{TFPD}\%\times \text{AAS}\\ \text{In Vitro PDCAAS}\;1=\mathrm{IV}-\text{PHDPD}\%\times \text{AAS}\\ \text{In Vitro PDCAAS}\;2=\mathrm{IV}-\text{PHSPD}\%\times \text{AAS}\\ \text{In Vitro PDCAAS}\;3=\mathrm{IV}-\text{SGPD}\%\times \text{AAS}\\ \text{In Vitro PDCAAS}\;4=\mathrm{IV}-\text{TIMPD}\%\times \mathrm{AAS} \end{array}$$

### 2.9 Statistical analysis

Data for composite samples are presented as mean plus the standard error of triplicate analyses. Data for all in vitro protein digestibility assays were corrected for casein digestibility values obtained within the same assay, assuming casein protein digestibility was 100%. Data were subjected to one‐way ANOVA using Tukey's multiple‐comparison test to assess significant differences between cooking methods (GraphPad Prism 9, GraphPad Software, LLC). A *p*‐value < .05 was chosen to indicate statistical significance. Data for the fried samples (raw [0], 3‐, 6‐, and 9‐min frying times) for AAS, in vitro protein digestibility, and in vitro PDCAAS were subjected to regression analysis (GraphPad) in order to determine the impact of frying time on in vitro protein quality measures.

## 3 RESULTS AND DISCUSSION

The dry matter, crude protein, and crude fat values for all processed Russet potatoes, represented on an “as consumed basis”, are presented in Table 1. Crude protein values were significantly higher for all of the cooking methods tested, relative to raw potato, with the exception of the boiled samples. Crude protein also increased significantly as frying time increased (fried 3 min [3.07 ± 0.07/100 g] to fried 9 min [4.77 ± 0.08/100 g]). The changes in protein content likely reflect the changes in moisture content during cooking, as all of the methods, with the exception of boiling, create conditions that will lead to the removal of water via evaporative losses, leading to an increase in the dry matter content. Expressing the nutrient content on an “as consumed basis” is nutritionally relevant for consumers. Additionally, all of the fried treatments resulted in a significant increase in fat when compared to unprocessed potatoes, or the other cooking treatments. The proximate values for the raw and cooked potatoes are consistent with values published in Food Data Central (U.S. Department of Agriculture, 2019). For example, the crude protein and fat content of restaurant French fries (NDB36014; Health Canada, 2023), a commodity generally made with Russet potatoes, were reported to be 3.49 and 14.0/100 g, respectively, consistent with the data observed in the current study for potatoes fried for 6 to 9 min. With respect to the protein composition, from a nutritional standpoint, the quality of the protein is particularly important. This is defined typically by understanding the impacts of cooking methods on amino acid content and protein digestibility.

**TABLE 1:** Nutrient composition (%) of Russet potatoes subjected to different consumer‐based cooking methods. Data are presented on an “as consumed” basis (fresh weight)^1^.

	Units	Raw (FDC^2^)	Raw	Microwaved	Baked	Boiled	Fried 3 min	Fried 6 min	Fried 9 min
Dry matter	%	20.8	21.39^f^ (0.23)	29.38^d^ (0.31)	24.77^e^ (0.083)	19.15^g^ (0.18)	41.8^c^ (0.71)	51.79^b^ (0.37)	63.93^a^ (0.39)
Crude fat	%	0.09	0.05^d^ (0.02)	0.06^d^ (0.02)	0.09^d^ (0.01)	0.07^d^ (0.01)	7.96^c^ (0.11)	9.99^b^ (0.08)	12.21^a^ (0.24)
Crude protein	%	2.05	1.85^e^ (0.04)	2.99^c^ (0.06)	2.44^d^ (0.03)	1.67^e^ (0.04)	3.07^c^ (0.07)	3.87^b^ (0.03)	4.77^a^ (0.08)
Alanine	%	0.063	0.048^d^ (0.0002)	0.047^d^ (0.0001)	0.047^d^ (0.0007)	0.025^e^ (0.0004)	0.059^c^ (0.0012)	0.067^b^ (0.0015)	0.075^a^ (0.0030)
Arginine	%	0.101	0.082^e^ (0.0022)	0.112^d^ (0.0024)	0.102^d^ (0.0014)	0.070^e^ (0.0013)	0.130^c^ (0.0015)	0.161^b^ (0.0023)	0.176^a^ (0.0049)
Aspartate	%	0.480	0.483^e^ (0.0116)	0.706^c^ (0.0116)	0.641^d^ (0.0136)	0.393^f^ (0.0092)	0.748^c^ (0.0158)	0.898^b^ (0.0060)	0.993^a^ (0.0078)
Cysteine	%	0.024	0.023^c^ (0.0001)	0.032^b^ (0.0009)	0.027^c^ (0.0008)	0.021^c^ (0.0002)	0.034^b^ (0.0012)	0.043^a^ (0.0005)	0.045^a^ (0.0010)
Glutamate	%	0.351	0.272^f^ (0.0114)	0.440^d^ (0.0014)	0.391^e^ (0.0047)	0.246^f^ (0.0051)	0.515^c^ (0.0017)	0.628^b^ (0.0128)	0.714^a^ (0.0149)
Glycine	%	0.057	0.045^e^ (0.0021)	0.062^c^ (0.0013)	0.054^d^ (0.0006)	0.041^e^ (0.0005)	0.067^c^ (0.0025)	0.083^b^ (0.0020)	0.101^a^ (0.0020)
Histidine	%	0.035	0.023^e^ (0.0010)	0.032^cd^ (0.0007)	0.029^d^ (0.0006)	0.021^e^ (0.0005)	0.036^bc^ (0.0010)	0.041^a^ (0.0011)	0.039^ab^ (0.0017)
Isoleucine	%	0.066	0.055^e^ (0.0005)	0.078^c^ (0.0025)	0.066^d^ (0.0009)	0.050^e^ (0.0005)	0.086^c^ (0.0023)	0.109^b^ (0.0021)	0.124^a^ (0.0019)
Leucine	%	0.098	0.085^d^ (0.0003)	0.119^c^ (0.0030)	0.097^d^ (0.0026)	0.080^e^ (0.0005)	0.127^c^ (0.0014)	0.160^b^ (0.0016)	0.192^a^ (0.0031)
Lysine	%	0.107	0.093^f^ (0.0007)	0.132^d^ (0.0032)	0.109^e^ (0.0020)	0.085^f^ (0.0011)	0.143^c^ (0.0023)	0.173^b^ (0.0016)	0.189^a^ (0.0036)
Methionine	%	0.032	0.034^d^ (0.0002)	0.047^c^ (0.0024)	0.041^c^ (0.0005)	0.029^d^ (0.0006)	0.055^b^ (0.0014)	0.069^a^ (0.0017)	0.068^a^ (0.0014)
Phenylalanine	%	0.081	0.067^f^ (0.0011)	0.098^d^ (0.0019)	0.084^e^ (0.0004)	0.062^f^ (0.0006)	0.110^c^ (0.0007)	0.138^b^ (0.0022)	0.161^a^ (0.0046)
Proline	%	0.063	0.048^f^ (0.0000)	0.069^d^ (0.0017)	0.058^e^ (0.0009)	0.045^f^ (0.0002)	0.077^c^ (0.0034)	0.093^b^ (0.0018)	0.111^a^ (0.0014)
Serine	%	0.074	0.062^de^ (0.0007)	0.086^cd^ (0.0016)	0.075^d^ (0.0022)	0.057^e^ (0.0011)	0.097^c^ (0.0014)	0.121^b^ (0.0023)	0.150^a^ (0.0067)
Threonine	%	0.067	0.053^e^ (0.0004)	0.076^c^ (0.0020)	0.065^d^ (0.0006)	0.049^e^ (0.0003)	0.081^c^ (0.0027)	0.100^b^ (0.0012)	0.120^a^ (0.0015)
Tryptophan	%	0.021	0.019^d^ (0.0005)	0.026^bcd^ (0.0035)	0.020^cd^ (0.0039)	0.017^d^ (0.0004)	0.031^abc^ (0.0003)	0.037^ab^ (0.0005)	0.041^a^ (0.0031)
Tyrosine	%	0.048	0.054^d^ (0.0025)	0.080^c^ (0.0017)	0.075^c^ (0.0011)	0.049^d^ (0.0013)	0.095^b^ (0.0024)	0.118^a^ (0.0031)	0.129^a^ (0.0059)
Valine	%	0.103	0.086^e^ (0.0009)	0.125^d^ (0.0024)	0.112^d^ (0.0015)	0.077^e^ (0.0006)	0.140^c^ (0.0030)	0.171^b^ (0.0044)	0.195^a^ (0.0032)
Amino acid score		0.724	0.660^ab^ (0.0100)	0.589^ab^ (0.0080)	0.569^b^ (0.0450)	0.675^a^ (0.0030)	0.474^c^ (0.009)	0.444^cd^ (0.012)	0.360^d^ (0.015)
First limiting amino acid		Leu	His	His	Leu	His	His	His	His

Abbreviations: His, histidine; Leu, leucine.

^1^ Data are presented as means, with standard error presented in brackets (*n* = 3).

^2^ Raw (USDA Food Data Central, NDB 11352) indicates data for raw potatoes, USDA Food Data Central. Data were subjected to one‐way ANOVA, and values with different superscripts are significantly different from each other (*p* < .05) as assessed by Tukey's procedure.

The amino acid compositions of the raw and cooked Russet potato samples are given in Table 1, expressed in % by weight. Consistent with data published in both Food Data Central (U.S. Department of Agriculture, 2019) and the Canadian Nutrient File (Health Canada, 2023), all the potato treatments contained the full spectrum of amino acids, including all indispensable amino acids. In general, changes in the amino acid contents of the raw and cooked potato samples mirrored changes in total protein content. In general, the protein and amino acid content of Russet potatoes demonstrated the following pattern (highest to lowest): Fried 9 min > Fried 6 min > Fried 3 min = Microwaved > Baking = Raw > Boiling. The latter changes likely reflect the overall removal of the dilution effect of moisture of the potatoes subjected to frying, microwaving, or baking, but not boiling.

Approximately 46% of the total amino acids was comprised of aspartate (28%) and glutamate (18%) across all treatments. Potatoes are known to possess significant amounts of nonprotein nitrogen (Hou et al., 2019; Kapoor et al., 1975), with estimates ranging from 40% to 70% (Kapoor & Li, 1983), depending on the methods used. Previous research has documented that the free amino acids asparagine and glutamine constitute approximately 65% of the total nonprotein nitrogen found in a cultivar (Red Pontiac) of *Solanum tuberosum* (Kapoor et al., 1975). Hou et al. (2019) have documented that free amino acids constitute 34.4% of total amino acids in potatoes, with asparagine being the most abundant (32.3%). During acid hydrolysis, as was done in this study to determine the total amino acid content (total of protein and nonprotein amino acids), the amino acids asparagine and glutamine are deamidated to yield, respectively, aspartate and glutamate. As such, the high proportion of the latter amino acids, particularly aspartate, in the raw and cooked potato samples is likely due to the presence of high amounts of free asparagine and, to a lesser extent, glutamine in the samples.

The absolute quantities of amino acids within a food, such as potato, are important for understanding their nutritional contribution to the human diet. However, understanding the quality of that protein is of critical importance. In Canada and the United States, protein quality is defined by the PDCAAS, a product of the AAS, a measure of how well the amino acids contained in the protein match against reference requirement patterns, and an in vivo measure of digestibility. With respect to the AAS (Table 1), using the reference pattern for 2‐ to 5‐year‐old school children, from the FAO/WHO, 1991 report (FAO/WHO, 1991), as required by the US Food and Drug Administration, the limiting amino acid in raw potato was histidine (0.66), followed by leucine. The cooking methods tested tended to lead to a reduction in the AAS (*p* < .05), except for boiling, with histidine remaining the limiting amino acid for all methods, except “Baked.” For the latter, leucine became the limiting amino acid. Histidine is sensitive to oxidation and can be converted into 2‐oxo‐histidine (Davies, 2005). As such, while this remains to be determined, reductions in the proportion of histidine relative to total protein may be a result of its oxidation during thermal processing, particularly those involving temperatures greater than 100°C, as boiling did not affect histidine levels. 2‐Oxo‐histidine was not included in the standard amino acid solutions involved in calibration, therefore additional insights will require studies specifically designed to test the hypothesis related to high‐temperature processing and histidine oxidation. Given the importance of the indispensable amino acid histidine as a dietary precursor for such pathways as one‐carbon (folate) metabolism and skeletal carnosine formation (Brosnan & Brosnan, 2020), the limiting nature of this amino acid in potatoes may require attention and consideration of complementary dietary sources.

As the plant‐based protein market expands, considerable attention has been paid to protein isolates derived from plant‐based sources as new ingredients in food and beverage formulations. Potato protein isolates have been positioned in the marketplace, and their amino acid composition generally, as assessed primarily by reviewing the AAS, is greater than 1.0. For example, Donadelli et al. (2019) reported the amino acid composition of several protein sources for potential use in pet foods, including the composition of a potato protein isolate. Calculation of the AAS from this data set yielded a value of 1.28, with histidine being the lowest ratio (albeit not limiting). During protein isolation, the protein‐bound nitrogen is separated from the nonprotein nitrogen, as such the final amino acid composition of the isolates would be enhanced due to removal of the dilution effect of free asparagine and glutamine. Further exploration of the impact of cooking methods on potato protein quality necessitates consideration of the digestibility of the protein.

For official analyses of PDCAAS, the TFPD% assay is required, particularly if claims related to protein are to be positioned on nutrition labels. In the current study, the TFPD% of raw potatoes was low at 40.5% (Table 2). This low level of protein digestibility may be related to the presence of certain antinutritive factors in raw potatoes, including alkaloids and lectins (Woolfe, 1987). Tuśnio et al. (2013) studied the impact of added potato protein concentrate with a focus on graded increases in solanidine glycoalkaloids and trypsin inhibitor activity on overall growth and protein digestibility in rats. They observed decreases in both study parameters, with subsequent experiments implicating the trypsin inhibitor activity as the primary suppressant (Tuśnio et al., 2013). Furthermore, raw potato starch consumption by rats has been shown to increase fecal weight by threefold (Calvert et al., 1989). Increased intake of the resistant starch fraction in raw potatoes may have led to an increase in microbial protein synthesis, with subsequent reductions in protein digestibility values. However, cooking clearly improves the overall protein digestibility, as values were increased above 80% for all of the cooking methods employed. For the in vivo assays, the “Frying” component was limited to a single evaluation of the treatment for 6 min, in order to reduce the number of animals under test. This level of protein digestibility is consistent with other grains and pulses subjected to various processing steps. For example, the TFPD% of thermally processed chickpeas ranged from 84.6% to 87.1% (Nosworthy et al., 2020), while that of various bean classes ranged from a low of 63.6% (baked black beans) to a high of 88.6% (baked faba beans) (Nosworthy et al., 2018b). Literature values for cooked potato protein digestibility are limited. A protein digestibility coefficient of 89% was cited in the FAO database on biological values for protein (FAO, 1970), with this coefficient derived from a study conducted approximately 100 years ago (Mitchell, 1924). There is a dearth of data related to whole potato protein digestibility, with most research focused on assessing the quality of potato protein concentrates or isolates. For the latter, Nestares et al. (1993) measured the TFPD% of a potato protein concentrate (84.6% protein) and determined it to be 93.3%, higher than that observed in the current study for the cooked whole potato treatments. As such, it is likely that constituents within the whole potato are having a suppressive effect on TFPD%.

**TABLE 2:** In vivo protein digestibility‐corrected amino acid score % (PDCAAS) of Russet potatoes subjected to different consumer‐based cooking methods^1^.

	Raw	Microwaved	Baked	Boiled	Fried 6 min
AAS^2^	0.660 (0.0100)	0.589 (0.0080)	0.596 (0.0450)	0.675 (0.0030)	0.444 (0.012)
True fecal protein digestibility (TFPD%)	40.5^b^ (3.92)	82.9^a^ (0.59)	84.5^a^ (0.49)	83.0^a^ (0.65)	80.1^a^ (1.59)
PDCAAS%	26.7^d^ (2.58)	48.8^b^ (0.35)	50.4^b^ (0.29)	56.0^a^ (0.44)	35.6^c^ (0.018)

^1^ Data for TFPD% are presented as means, with standard Error presented in brackets (*n* = 10). Data for PDCAAS were calculated as the product of the mean AAS and the individual TFPD% replicate values. Data were subjected to one‐way ANOVA, and Tukey's multiple‐comparison test was used to assess differences between treatment means. Values with different superscripts are significantly different from each other (*p* < .05).

^2^ Data for AASs are repeated from Table 1.

The PDCAAS values for the raw and cooked Russet potato treatments are given in Table 2. Given the low TFPD% for the raw control treatment, the PDCAAS was found to be 0.27. This value is lower than certain raw plant‐based proteins, including wheat (0.42), oats (0.69), and soy flour (0.75) (Nosworthy et al., 2023). However, in studies designed to measure the impact of cooking on the protein quality of pulses (Nosworthy et al., 2018a, 2018b, 2020; Nosworthy, Neufeld, et al., 2017), it was not possible to determine the in vivo PDCAAS as the rats would not consume the diets based on raw pulses, thus eliminating the opportunity to derive TFPD% measurements. The latter studies agree with the current research in that cooking significantly enhanced TFPD% and led to significantly higher PDCAAS (%) for all of the cooked potato treatments, with boiled potatoes having the highest PDCAAS (%). The observed values, ranging from 0.488 to 0.560, are greater than those observed for cooked wheat (0.388–0.405; Nosworthy et al., 2023 and comparable to values observed for roasted almonds 0.443–0.478; House et al., 2019). The profile of limiting amino acids in the protein of Russet potatoes, with limitations in histidine and leucine, makes this protein source complementary to pulses (navy beans = 0.667; split yellow peas = 0.643; chickpeas = 0.519; and green lentils = 0.628), as the latter tend to be deficient in either the sulfur amino acids or tryptophan (Nosworthy, Medina, et al., 2017).

A major challenge to understanding the impact of processing conditions, as well as other sources of variability, including crop genetics, environmental, and management factors, with respect to protein quality lies in the use of an in vivo animal model or bioassay to determine protein digestibility. The availability of alternative models based on in vitro techniques provides an opportunity to assess factors influencing protein digestibility more rapidly. For the current study, we employed four in vitro models, and the data are given in Table 3. All the in vitro methods overestimated the in vivo digestibility of protein in the raw control, as values ranged from 73.1% to 80.0% versus the 40.5% observed for the TFPD%. In general, the IV‐PHDPD% values yielded the lowest values, with responses that were somewhat blunted in comparison to the other in vitro techniques. For the latter method, microwaving, baking, boiling, and 9‐min frying yielded digestibility coefficients that were significantly lower than the raw control. The IV‐PHSPD% method did not yield significant differences between cooking methods for in vitro protein digestibility. However, the IV‐SGPD% and the IV‐TIMPD% methods provided evidence that raw Russet potato had the lowest in vitro protein digestibility coefficients, consistent with the in vivo TFPD% data.

**TABLE 3:** In vitro protein digestibility^1^ of Russet potatoes subjected to different consumer‐based cooking methods^2^.

In vitro digestibility method^3^	Raw	Microwaved	Baked	Boiled	Fried 3 min	Fried 6 min	Fried 9 min
IV‐PHDPD%	78.7^a^ (0.28)	76.9^bc^ (0.31)	76.5^c^ (0.31)	76.5^c^ (0.27)	77.3^abc^ (0.10)	78.3^ab^ (0.53)	76.2^c^ (0.47)
IV‐PHSPD%	74.1 (0.61)	78.1 (2.27)	79.2 (0.98)	74.3 (0.53)	78.4 (0.58)	78.0 (0.71)	76.6 (1.72)
IV‐SGPD%	73.1^b^ (0.28)	86.3^a^ (1.98)	87.8^a^ (1.59)	86.0^a^ (2.02)	84.9^a^ (4.58)	91.1^a^ (2.52)	85.8^a^ (1.89)
IV‐TIMPD%	80.0^b^ (3.46)	95.1^a^ (4.63)	91.0^ab^ (1.76)	92.5^ab^ (2.86)	96.4^a^ (2.03)	94.5^a^ (2.12)	91.9^ab^ (0.27)

^1^ In vitro protein digestibility values (corrected for casein = 100%) are shown.

^2^ Data are presented as means, with standard error presented in brackets (*n* = 3). Data were subjected to one‐way ANOVA, and Tukey's multiple‐comparison test was used to assess differences between cooking methods. Values with different superscripts are significantly different from each other (*p* < .05).

^3^ The in vitro protein digestibility procedures were as follows: IV=PHDPD = pH‐Drop procedure; IV‐PHSPD = pH‐Stat procedure; IV‐SGPD = simulated gastrointestinal digestion procedure; IV‐TIMPD = dynamic gastric digestion model (TIM‐1).

The IV‐PHPD% and IV‐PHSPD% methods represent a lower level of complexity in relation to modeling digestive processes. The use of the IV‐SGDPD% method represents a step up in complexity in that, while still being a static method, it employs a two‐stage digestion protocol (gastric and intestinal), with the addition of α‐amylase. Nu et al. (2020) used a similar method to compare the in vitro protein digestibility of conventional and thermomechanical and enzyme‐facilitated processed soybean meal for pigs and determined that the advanced processing methods used for soybean preparation yielded a product with higher IVPD (74.6% vs. 81.1% at 6 h total digestion). These authors also demonstrated that the differences in IVPD values were consistent with differences observed for standardized ileal digestible coefficients for crude protein, being 88.4% and 90.3% (*p* < .001) for the conventional and advanced processed soybean meal, as measured in an in vivo pig study (Nu et al., 2020). In the current study, the IV‐SGPD% values for all the cooked potato samples were significantly different from the raw control (Table 3), with all methods being higher than the control. Like the other static in vitro methods, the digestibility coefficient for the raw control overestimated the in vivo TFPD% measure. The advantages of the two‐stage IV‐SGPD% method include the fact that, for a static method, it better reflects gastrointestinal digestion. Recently, efforts have been placed, internationally, to standardize an in vitro model to simulate human digestion (INFOGEST; Brodkorb et al., 2019). At the time when the current experiments were conducted, the INFOGEST protocol was not standardized for measuring protein/amino acid digestibility, thus efforts were placed on the use of more established techniques. Recently, the INFOGEST protocol has been used to determine the in vitro digestible indispensable amino acid score (DIAAS) for several food items, but not potatoes (Sousa, Portmann, et al., 2023; Sousa, Recio, et al., 2023), and future efforts should be placed toward advancing this standardized methodology. A potential limitation of the IV‐SGPD% method, as applied in the current study, relates to the use of OPA as a reagent for measuring *N* release from potato protein. It is recognized that OPA has limited reactivity with certain amino acids, including proline and cysteine, which may have had an impact on the final in vitro protein digestibility values. Regardless, the use of the IV‐SGPD% method in the current study was sufficiently sensitive to position relative differences due to cooking methods that were consistent with in vivo observations.

Advancing the level of complexity for in vitro protein digestibility methods led to the use of the dynamic TIM‐1 system. This method involves the use of a proprietary, computer‐controlled artificial stomach and small intestinal tract (Minekus, 2015). This method has been used to measure the in vitro protein digestion kinetics of milk proteins (Abrahamse et al., 2022), the digestibility of various plant proteins subjected to dietary probiotics (Keller et al., 2017), as well as short‐dough biscuits enriched in proteins (Villemejane et al., 2016). Havenaar et al. (2016) measured true ileal digestibility coefficients of immature herring egg protein, and determined that the values were correlated with human ileal digestibility values (*r* ^2^ = 0.96), with digestibility values ranging from 71% to 92%. In the current study, the IV‐TIMPD% followed the same pattern as the static in vitro methods, with all methods of cooking yielding digestibility values that were higher and significantly different from the raw control. For the current set of experiments, the relative differences versus the raw control observed with the dynamic method as compared to the three static methods did not specifically confer an advantage for the former method.

**FIGURE 1:**
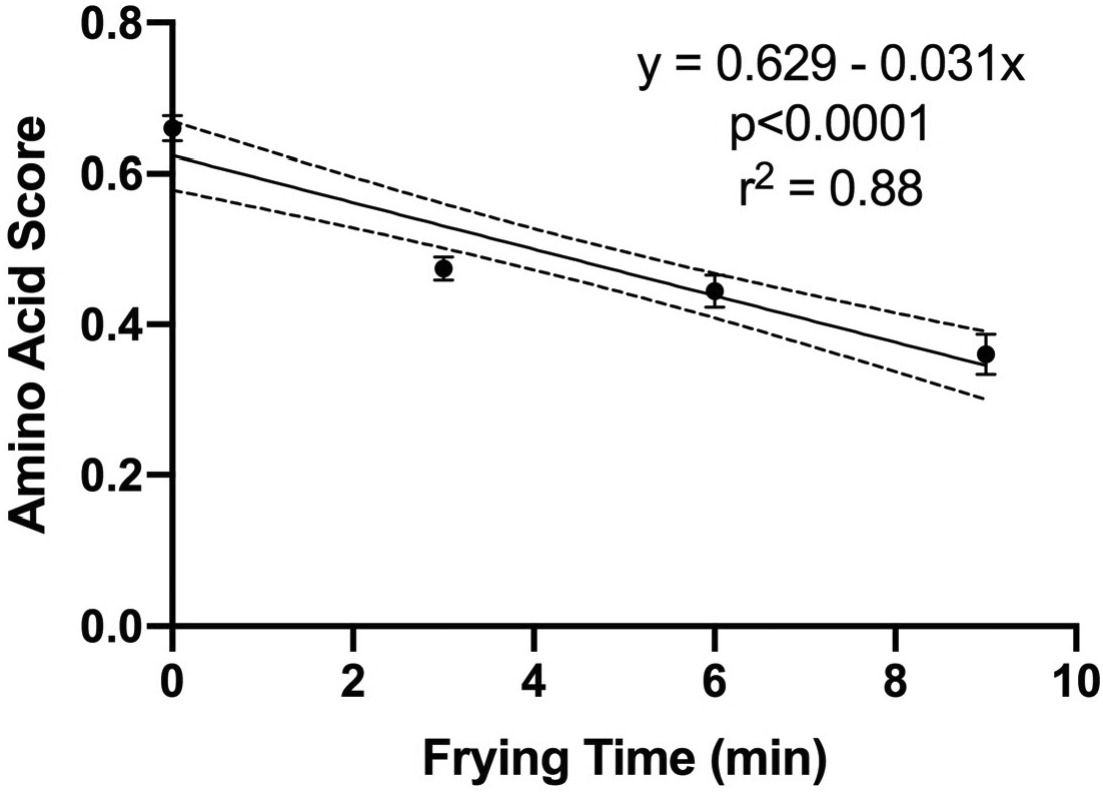
The impact of deep oil frying time (minutes) at 190°C on the amino acid score of Russet potatoes. Data are presented as means ± SEM (*n* = 3) with 95% confidence intervals.

The data for all the in vitro PDCAAS values, as measured with the four different methods, are given in Table 4. The final calculated values, representing the product of the AAS (Table 1) and the in vitro digestibility coefficients (Table 3), are primarily influenced by the relative changes in the AAS, particularly given the overestimation by all of the in vitro methods of the TFPD% of the raw control. In general, the greatest differences observed against the control appeared to be with the fried samples. As such, the data for AAS, in vitro protein digestibility, and final in vitro PDCAAS were subjected to regression analysis, exploring the relationship between the duration of frying time and the in vitro measures of protein quality. As depicted in Figure 1, there was a negative linear relationship between frying time and AAS (*r* ^2^ = 0.88; *p* < .0001). As the AAS was driven by the histidine content of the samples (Table 1), these data further support the potential susceptibility of this amino acid to destruction, potentially due to oxidation generated during the high‐temperature frying conditions. The protein digestibility data for the four in vitro models were fitted to second‐order polynomial equations (Table S1), with varying goodness of fit. The simple static methods (IV‐PHDPD%, *r* ^2^ = 0.44; IV‐PHSPD%, *r* ^2^ = 0.54) fit the nonlinear regression model to a lesser extent, as compared to the IV‐SGPD% (*r* ^2^ = 0.73) and the IV‐TIMPD% (*r* ^2^ = 0.72) methods. The latter two methods highlighted increased digestibility to a peak following 6 min of frying with a subsequent decrease as frying time increased to 9 min. Increasing frying time has been shown to lead to a decrease in the “L” value of Russet potatoes (Abduh et al., 2021). This increasing darkness is a function of Maillard reaction products which can depress the digestibility of food proteins (Zenker et al., (2020), potentially explaining the later reduction observed in the IV‐SGPD% and IV‐TIMPD% at 9 min of frying. Similar to the AAS regression data, the final IV‐PDCAAS values for the four in vitro methods exhibited a negative linear relationship as a function of frying time (Table S1), with *r* ^2^ values ranging between 0.76 and 0.90. Clearly, extended durations of oil frying suppress the protein quality of Russet potatoes due principally to a reduction in the AAS.

**TABLE 4:** In vitro protein digestibility‐corrected amino acid score (%)^1^ of Russet potatoes subjected to different consumer‐based cooking methods^2^.

	Raw	Microwaved	Baked	Boiled	Fried 3 min	Fried 6 min	Fried 9 min
IV‐PDCAAS1	51.9^a^ (0.19)	45.3^b^ (0.18)	43.6^c^ (0.18)	51.6^a^ (0.18)	36.7^d^ (0.05)	34.8^e^ (0.23)	27.5^f^ (0.17)
IV‐PDCAAS2	48.9^ab^ (0.40)	46.0^b^ (1.34)	45.1^b^ (0.56)	50.2^a^ (0.36)	37.2^c^ (0.28)	34.6^c^ (0.31)	27.6^d^ (0.62)
IV‐PDCAAS3	48.2^b^ (0.19)	50.9^b^ (1.17)	49.9^b^ (0.91)	58.1^a^ (1.36)	40.2^c^ (2.17)	40.7^c^ (1.12)	30.9^d^ (0.68)
IV‐PDCAAS4	52.8^bc^ (2.29)	56.0^ab^ (2.72)	51.8^bc^ (1.00)	62.4^a^ (1.93)	45.7^cd^ (0.96)	42.0^d^ (0.94)	33.1^e^ (0.10)

^1^ In vitro protein digestibility corrected amino acid score % (IV‐PDCAAS) calculated as the product of AAS (Table 1) and the respective in vitro protein digestibility method (Table 3): IV‐PDCAAS1 = AAS × IV‐PHDPD%; IV‐PDCAAS2 = AAS × IV‐PHSPD%; IV‐PDCAAS3 = AAS × IV‐SGPD%; IV‐PDCAAS4 = AAS × IV‐TIMPD%.

^2^ Data were subjected to one‐way ANOVA, and Tukey's multiple‐comparison test was used to assess differences between treatment means. Values with different superscripts are significantly different from each other (*p* < .05).

## 4 CONCLUSION

The current study measured the impact of various cooking methods on the protein quality of Russet potatoes represented in a whole‐food, not isolated, form. This study demonstrated that cooking changes both the AAS and digestibility coefficients, with results varying as a function of both the cooking method and the method used to measure protein digestibility. With respect to the AAS, the high‐moisture boiling methods were protective of the limiting amino acids, whereas all other cooking methods led to reductions in the limiting amino acid histidine, with concomitant reductions in the AAS. Frying was particularly damaging to the AAS. However, protein digestibility was dramatically improved with cooking, as was observed with the in vivo TFPD% method and the more sophisticated in vitro methods (static: IV‐SGPD%; dynamic: IV‐TIMPD%). The final PDCAAS values, both in vivo and in vitro, mirrored the changes in the AAS.

Based on the current PDCAAS estimates for potato protein, pulses such as beans, peas, chickpeas, and lentils could serve as suitable complementary protein sources to balance amino acid supply in the human diet. The findings from this study offer valuable information for modeling the protein quality of the human diet and assessing the role of potatoes in meeting dietary protein requirements. If viewed through a lens of protein quality, efforts should be placed to encourage the transition towards cooking methods such as baking or boiling, over frying, to enhance the intake of quality‐corrected protein for consumers, and their consumption with complementary protein sources (e.g., navy beans). These approaches align with recent studies that have investigated protein sources in the Canadian diet and their quality (Marinangeli et al., 2021).

## AUTHOR CONTRIBUTIONS

**Taryn Bailey:** Conceptualization (equal); data curation (equal); formal analysis (equal); investigation (lead); methodology (lead); writing – original draft (equal). **Adam J. Franczyk:** Formal analysis (equal); methodology (equal); supervision (supporting). **Erin M. Goldberg:** Formal analysis (supporting); writing – review and editing (equal). **James D. House:** Conceptualization (lead); data curation (lead); formal analysis (equal); funding acquisition (lead); investigation (supporting); methodology (supporting); project administration (lead); resources (lead); supervision (lead); validation (lead); visualization (equal); writing – original draft (equal); writing – review and editing (equal).

## CONFLICT OF INTEREST STATEMENT

The authors declare that they do not have any conflicts of interest.

## ETHICS STATEMENT

All animal procedures received approval from the University of Manitoba's Institutional Animal Care Committee, which utilizes the appropriate guidelines established by the Canadian Council on Animal Care (2018).

## Supporting information


Table S1.

## Data Availability

The data that support the findings of this study are available on request from the corresponding author.
