# Supplementary material for: Impact of cooking on the protein quality of Russet potatoes
Source: Food Sci Nutr. 2023 Oct 3;11(12):8131–42. doi: 10.1002/fsn3.3734 (PMC10724643; doi:10.1002/fsn3.3734)
Supplement: Supplementary file 1 — Table S1. [file FSN3-11-8131-s001.docx]

***Supplementary Table 1***. Regression analysis of the impact of deep oil frying on the *in vitro* digestibility of protein contained in Russet potatoes^1^.

| ***In Vitro* Protein Digestibility Method^2^** | **Equation**  Y = B0 + (B1)(X) + (B2)(X^2^) | | | r^2^ |
| --- | --- | --- | --- | --- |
|  | B0 | B1 | B2 |  |
| IV-PHDPD % | 78.40  (77.21; 79.59) | -0.0293  (-0.667; 0.609) | -0.020  (-0.088; 0.048) | 0.44 |
| IV-PHSPD% | 74.31  (72.11; 76.52) | 1.643  (0.464; 2.823) | -0.156  (-0.282; -0.031) | 0.54 |
| IV-SGPD% | 72.67  (66.74; 78.59) | 5.942  (2.770; 9.113) | -0.494  (-0.831; -0.156) | 0.73 |
| IV-TIMPD% | 80.87  (75.34; 86.41) | 5.874  (2.913; 8.835) | -0.528  (-0.843; -0.212) | 0.72 |

^1^Data are presented as Means, with 95% Confidence Intervals in parentheses. Data were fitted to a second order polynomial equation.

^2^The in vitro protein digestibility procedures were as follows: IV=PHDPD = pH Drop procedure; IV-PHSPD = pH-Stat procedure; IV-SGPD = simulated gastrointestinal digestion procedure; IV-TIMPD = dynamic gastric digestion model (TIM-1).
